# Microplastics Exposure Aggravates Synovitis and Pyroptosis in SLE by Activating NF-κB and NRF2/KEAP1 Signaling

**DOI:** 10.3390/toxics12120840

**Published:** 2024-11-22

**Authors:** Wenxiang Zeng, Shiqiao He, Ying Zhao, Minjian Jiang, Wenla Wang, Limeng Yang, Weibin Du, Wei Zhuang

**Affiliations:** 1 The Third Clinical Medical College, Zhejiang Chinese Medical University, Hangzhou 310053, China; 18070519359@163.com (W.Z.);; 2 Hangzhou Xiaoshan Hospital of Traditional Chinese Medicine, Hangzhou 311200, China; 3 The First Clinical Medical College, Zhejiang Chinese Medical University, Hangzhou 310053, China; 4 The First Affiliated Hospital of Zhejiang Chinese Medical University, Zhejiang Provincial Hospital of Chinese Medicine, Hangzhou 310006, China; 5 Jiaxing Hospital of Traditional Chinese Medicine, Jiaxing 314500, China

**Keywords:** *MRL*/*lpr* mice, microplastics, synovitis, oxidative stress, cell pyroptosis, NF-κB signaling

## Abstract

Microplastics (MPs) represent an emerging pollutant capable of entering the human body through the respiratory and digestive systems, thereby posing significant health risks. Systemic lupus erythematosus (SLE) is a complex autoimmune disease that affects multiple organ systems, often presenting with polyarticular joint manifestations. Despite its relevance, there is currently limited research on the impact of MPs on lupus arthritis. This study aims to investigate the effects of MPs on joint inflammation in SLE. *MRL*/*lpr* mice exhibit SLE similar to that of humans. We administered either 0.5 mg/kg or 5 mg/kg of MPs to 8-week-old female *MRL*/*lpr* mice via oral ingestion. Our findings indicate that exposure to MPs can lead to synovial damage, adversely affecting the morphology and function of the knee joint, along with increased oxidative stress, apoptosis, synovial fibrosis, and the secretion of inflammatory cytokines. Notably, MPs significantly enhanced synovial cell pyroptosis by upregulating the expression of NLRP3, CASPASE-1, GSDMD, IL-1β, and IL-18. Mechanistic analyses further demonstrated that MPs exposure activates the NF-κB and NRF2/KEAP1 signaling pathways. Overall, our in vivo findings suggest that MPs exposure promotes synovial cell pyroptosis through increased oxidative stress and NF-κB signaling, thereby disrupting the structure and function of synovial tissue. This research provides new insights into the synovial damage associated with MPs exposure.

## 1. Introduction

Since the 1950s, MPs with particle sizes smaller than five millimeters have been widely utilized. Over the decades, plastic fragments have accumulated in various locations worldwide, posing significant threats to ecological and human health [1,2]. Research has indicated extensive damage resulting from MPs exposure. For example, four-week-old mice subjected to MPs for 12 weeks before ischemia–reperfusion injury exhibited aggravated damage to renal tubules and glomeruli. MPs exposure substantially upregulated the expression of apoptosis-related genes, including NLRP3, GSDMD, CASPASE-1, and IL-18 [3]. Additionally, oral administration of MPs at a dosage of 5 mg/kg for 28 days resulted in liver inflammation, increased expression of IL-6 and TNF-α, disrupted lipid metabolism, and adverse effects on the intestines, leading to intestinal inflammation and compromised intestinal barrier integrity [4]. Overall, the effects of MPs exposure vary across different organs, including the liver, kidneys, intestines, testes, lungs, and muscles, depending on the concentration and duration of exposure [5].

The synovium, located within the joint cavities, lines the internal surface of the joint capsule and serves as a crucial barrier for molecular exchange between the joint cartilage and plasma [6]. It plays a vital role in the development of joint injuries and diseases, contributing to symptoms such as pain and swelling. Changes in joint tissues due to injuries and illnesses can also occur via the synovial fluid [7]. SLE is a complex autoimmune disease that affects multiple organs [8]. Studies have shown that symptomatic patients with SLE, even in the absence of clinically evident joint swelling, exhibit ultrasonographic synovitis involving two or more joints, characterized by joint swelling, effusion, or tenderness [9,10]. However, the changes in synovial tissue and the molecular mechanisms of injury following exposure to MPs in SLE mice remain unclear.

Cell pyroptosis is a form of programmed inflammatory cell death mediated by the gasdermin protein family. This process is characterized by the formation of transmembrane pores, cell swelling and rupture, and the release of various inflammatory cytokines and cytoplasmic contents [11,12]. Current evidence suggests that exposure to 5 μm MPs triggers cell pyroptosis through the NF-κB-NLRP3-GSDMD axis, exacerbating myocardial inflammatory responses with increased expression of NLRP3, CASPASE-1, IL-1β, IL-18, GSDMD, NF-κB [13]. Another study suggests that 0.5 μm MPs can induce both pyroptosis and apoptosis in ovarian granulosa cells through the NLRP3/caspase-1 signaling pathway, potentially triggered by oxidative stress [14]. However, pyroptosis of synovial cells after MPs exposure has not been reported, and we hypothesized that synovial cell pyroptosis may also be involved in MP-induced synovial damage.

Cell pyroptosis can be induced by various stimuli, including oxidative stress [13]. The NRF2/KEAP1 signaling pathway plays a critical role in regulating antioxidant defenses. Oxidative stress is closely associated with tissue damage resulting from exposure to MPs. Increasing evidence suggests that MPs exposure triggers oxidative stress in the intestine [15], ovaries [16], liver [17], and skeletal muscles [18]. The interaction between NRF2 and NF-κB is complex, as these pathways jointly regulate each other’s expression. Notably, the NRF2 pathway can attenuate the activity of NF-κB by elevating levels of antioxidants and cytoprotective enzymes [19]. However, it remains unclear whether exposure to MPs modulates the oxidative stress response through the NRF2/KEAP1 and NF-κB signaling pathways, potentially leading to synovial degeneration.

Given the potential toxicity of MPs reported in previous studies on the general population’s exposure [13,14], we selected doses of 0.5 mg/kg and 5 mg/kg as experimental levels to further investigate the mechanisms of MP-induced synovial damage. *MRL*/*lpr* mice are commonly used as SLE models. Due to the observed sex bias in humans and the delayed disease progression in male *MRL*/*lpr* mice [20], female mice were used in this study. A total of 10 female C57BL/6 J mice and 30 *MRL*/*lpr* mice (8 weeks old) received gastric administration of the two doses of MPs for 9 weeks. The assessment of synovial oxidative stress markers and synovial structure was conducted through histological staining, immunohistochemical analysis, and other methodologies.

## 2. Material and Methods

### 2.1. Reagents

Single-molecule microparticles (CAS: 79633), with a particle diameter of five micrometers, were purchased from Sigma-Aldrich, Inc. (St. Louis, MO, USA) Before the experiment begins, distilled water is used to prepare the desired concentration, and then the particles are subjected to ultrasonic treatment. The MASSON Staining kit was obtained from Solarbio (Beijing, China). The antibodies utilized in this study included MMP13, MMP19, and IL-6, which were sourced from HuaBio (Hangzhou, China); CASPASE-3, BCL-2, IL-18, P65, TNF-α and p-P65, which were purchased from Ruiying Biology (Shanghai, China); IL-1 β, acquired from Bioss (Beijing, China);NLRP3, CASPASE-1, and HO-1, obtained from Proteintech (Chicago, IL, USA); GSDMD, purchased from Abcam (Cambridge, UK); NRF2 and KEAP1, acquired from Immunoway (San Diego, CA, USA); and IκBα and p-IκBα, sourced from Beyotime (Shanghai, China). All the antibodies were proportioned according to the manufacturer’s instructions. The TUNEL apoptosis detection kit was purchased from Vazyme Biotech (Nanjing, China).

### 2.2. Animals

Seven-week-old female C57BL/6J and *MRL*/*lpr* spontaneous lupus-prone mice (20–25 g) were provided by the Animal Experiment Center of the Zhejiang Chinese Medical University. All the mice were housed in specific pathogen-free animal care facilities and placed in cages primarily made of polycarbonate. The room temperature was maintained at 23 ± 2 °C, with a relative humidity of 50%. The rooms operated under a 12 h light/dark cycle, and the mice had ad libitum access to water and laboratory feed. We try to minimize exposure to MPs in the environment or diet. The mice were acclimated to these living conditions for one week. All experiments were conducted following appropriate guidelines and regulations. Approval for all mouse procedures was obtained from the Animal Experiment Ethics Committee of the Zhejiang Chinese Medical University.

### 2.3. Experimental Design

**Figure 1:**
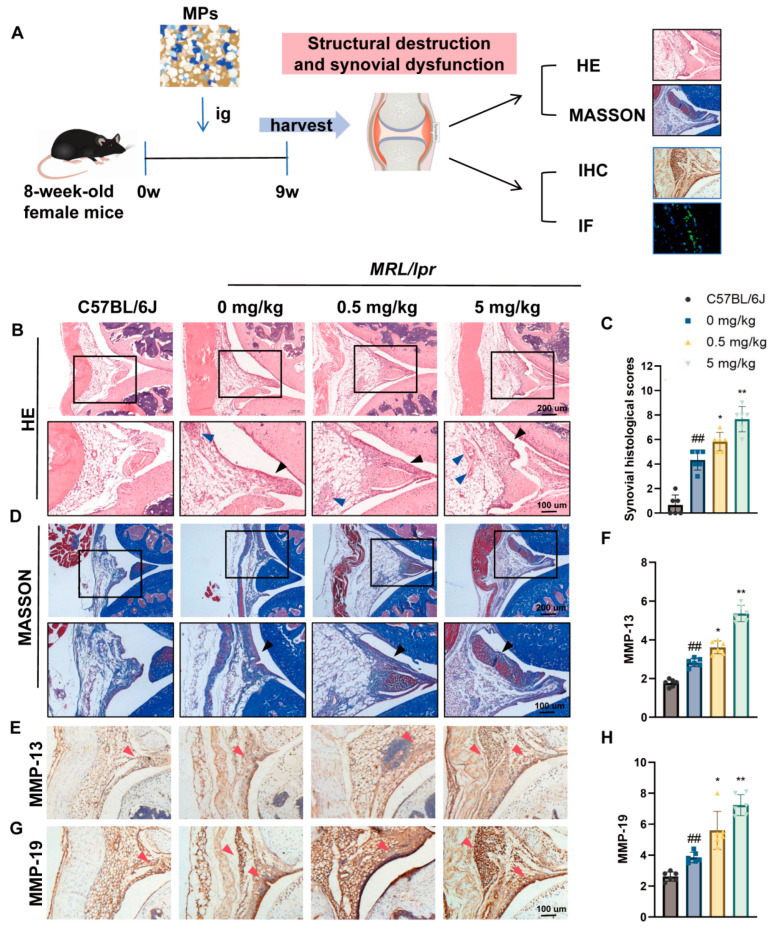
The MP structure damages the synovial structure and aggravate synovitis in SLE mice: (**A**) flow chart of the experimental design; (**B**) HE staining of mouse knee synovium, demonstrating thickening of synovial layers (Black arrow) and increased vasculature (Blue arrow); (**C**) histological scoring of the synovium based on HE staining; (**D**) MASSON’s trichrome staining of mouse knee synovium, indicating increased fiber content (Black arrow); (**E**–**H**) immunohistochemical staining illustrating the expression of MMP-13 and MMP-19 in the synovium of the knee joint of mice, along with quantification of their expression levels. Red arrowheads denote positively stained cells. Data are presented as mean ± standard deviation (SD). ns indicates no statistical significance. * *p* < 0.05, ** *p* < 0.01 (vs. 0 mg/kg group); ## *p* < 0.01 (vs. C57BL/6J group), with *n* = 6 per group.

Ten female C57BL/6J mice and 30 *MRL*/*lpr* mice were randomly divided into three groups (*n* = 10 per group): a control group, a low-dose microplastics (MPs) group (0.5 mg/kg), and a high-dose MPs group (5 mg/kg). The selection of MP dosage is based on previous studies [4,13] and is set according to the actual range of daily MPS intake for adults (0.2–10.2 mg/b.w./day) [21]. Mice in both the low-dose and high-dose MP groups were gavaged five times per week, while mice in the control group received an equivalent volume of water. After 9 weeks of treatment, euthanasia was performed using carbon dioxide. Subsequently, the skin around the knee joints of the mice was excised and the muscles and fascia were carefully cut using scissors. The femur and tibia were then sectioned at the midpoint, and the knee joints were removed (Figure 1A).

### 2.4. Histological Analyses

For histological analysis, mouse knee joint specimens were obtained. The specimens were initially fixed in 4% formaldehyde for 24 h, followed by a 4-week decalcification process using 14% EDTA (pH = 7.4). Subsequent steps included dehydration, paraffin embedding, and processing into 5 mm thick sagittal sections. Deparaffinization was performed using xylene, followed by rehydration with a series of decreasing ethanol concentrations. Morphological assessment was conducted through Hematoxylin and Eosin (HE) staining. Additionally, MASSON’s trichrome staining was performed according to the manufacturer’s protocol to analyze the degree of fibrosis in the synovium.

Histological scoring of the synovial tissue was then conducted based on the results of HE and MASSON’s staining, following a pre-established protocol [22]. Microscopic observations of synovial tissues from different groups were evaluated based on four criteria, with each criterion contributing to a four-level scoring system. The scores for each parameter were cumulatively assessed in each group, as detailed in Table 1.

### 2.5. Immunofluorescence (IF) and Immunohistochemical (IHC) Staining Analyses

Following specimen acquisition, decalcification was conducted using 14% EDTA (pH 7.4) for four weeks. Subsequently, the specimens were subjected to dehydration, paraffin embedding, and sectioning into 5 mm thick sagittal sections. Incubation with 5% normal goat serum was performed at room temperature for 1 h, followed by overnight incubation at 4 °C with the corresponding primary antibodies against MMP13, MMP19, CASPASE-3, BCL-2, IL-1β, IL-6, TNF-α, NLRP3, CASPASE-1, GSDMD, IL-18, NRF2, KEAP1, HO-1, P65, p-P65, IκBα, and p-IκBα (detailed information in Table 1). The next day, fluorescently labeled secondary antibodies were added and incubated at 37 °C in the dark for 1 h. Vector^®^ TrueVIEW^®^ autofluorescence quenching reagent was utilized to eliminate nonspecific fluorescence. Negative control sections were incubated with nonspecific IgG. Finally, DAPI counterstaining was performed, and images were captured using a fluorescence microscope (Carl Zeiss, Gottingen, Germany). For IHC, the secondary antibody was added the next day and incubated for 30 min, followed by DAB color development, hematoxylin staining of the nucleus, and neutral resin sealing. Based on the average fluorescence intensity and IHC average expression area, tissue morphological quantification was conducted using Image-Pro Plus 6.0.

### 2.6. TUNEL Assay

According to the manufacturer’s instructions, we use the TUNEL apoptosis detection kit to determine in situ apoptotic cells. We randomly select six samples from each group, and we use DAPI to identify cell nuclei and quantify the total number of apoptotic cells.

### 2.7. Statistics Analysis

All numerical data were presented as means ± SD. Statistical analysis was conducted using GraphPad Prism 9.5.0. Paired *t*-tests, Welch’s correction, and Mann–Whitney U tests were conducted to compare differences among groups. A *p*-value of less than 0.05 was considered statistically significant.

## 3. Results

### 3.1. MPs Damage Synovial Structure and Aggravate Synovial Inflammation in SLE Mice

To investigate the impact of MPs exposure on the morphological structure of synovial tissue, we conducted analyses using HE staining and MASSON’s trichrome staining. The results revealed that MPs exposure significantly heightened inflammation in the synovial tissue of SLE mice, characterized by notable tissue thickening, increased infiltration of inflammatory cells, enhanced vascular proliferation, and elevated histological scores for synovial tissue. After exposure to low-dose MP, histological scores can increase by 0–4 points, while high-dose MP can increase by 1–6 points (Figure 1B,C). Furthermore, MPs exposure resulted in a substantial increase in fibrous tissue proliferation at the periphery of the synovium (Figure 1D). The expression levels of matrix metalloproteinases MMP-13 and MMP-19, enzymes implicated in synovial matrix metabolism, were significantly elevated (Figure 1E–H). Additionally, no apparent dose-dependent relationship was observed regarding the structural damage to synovial tissue caused by MPs.

### 3.2. MPs Promote Synovial Cell Apoptosis in SLE Mice

**Figure 2:**
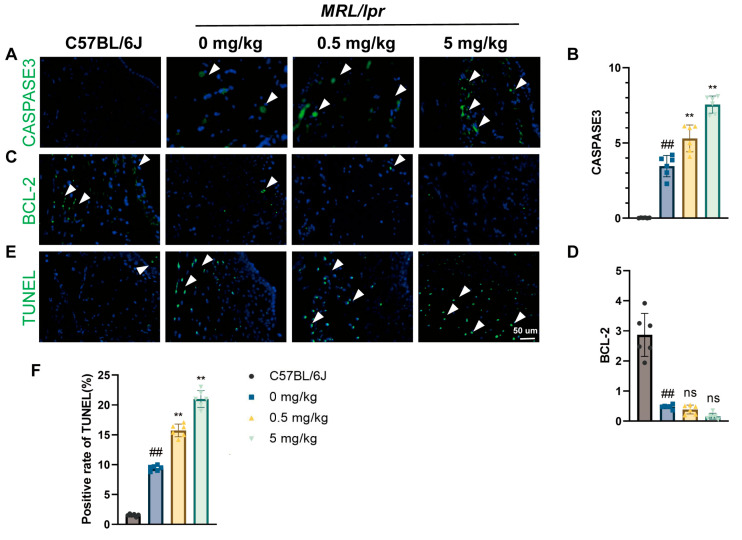
MPs promote synovial cell apoptosis in SLE mice: (**A**,**C**) immunofluorescence staining demonstrating the expression of CASPASE-3 and BCL-2 in the synovium of the knee joints of mice, along with (**B**,**D**) quantification of expression; (**E**) TUNEL staining and (**F**) quantification of the rate of TUNEL-positive cells. DAPI stains the nuclei blue, and white arrowheads indicate positively stained cells. Data are presented as mean ± SD. ns indicates no statistical significance. ** *p* < 0.01 (vs. 0 mg/kg group); ## *p* < 0.01 (vs. C57BL/6J group), *n* = 6 per group.

To evaluate the effect of MP exposure on synovial cell apoptosis, we assessed the expression of apoptosis markers through IF analysis. The results showed that MP exposure did not significantly alter the expression of anti-apoptotic protein BCL-2; however, it did enhance the expression of apoptotic protein CASPASE-3 in a dose-dependent manner (Figure 2A–D). Meanwhile, TUNEL staining further confirmed the pro apoptotic effect of MP. The TUNEL staining results showed that exposure to MP significantly increased the number of TUNEL-positive cells in synovial tissue, with higher doses being more pronounced. After exposure to low-dose MP, the apoptosis rate can increase by 5%, while high-dose MP can increase by 10% (Figure 2E,F). These findings indicate that MP can promote apoptosis of mouse synovial cells.

### 3.3. MPs Compound Synovial Inflammation in SLE Mice

**Figure 3:**
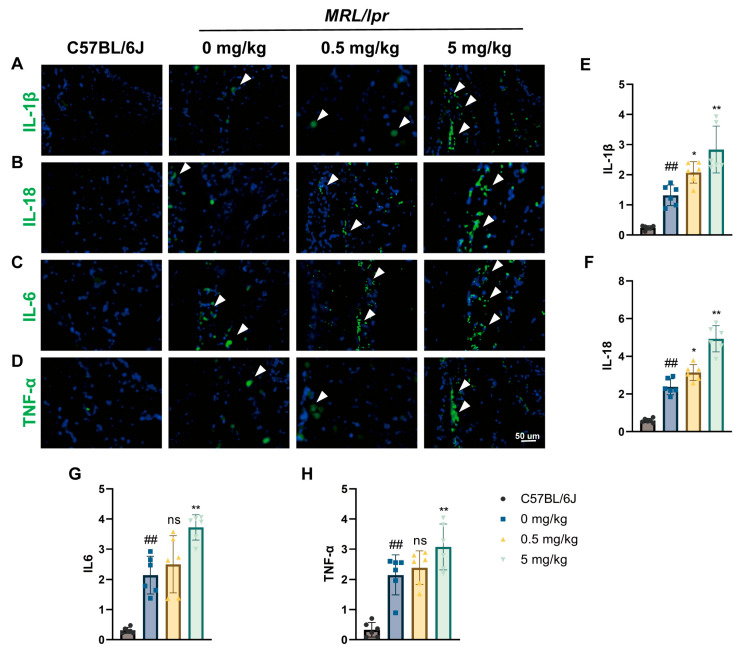
MPs compound synovial inflammation in SLE mice. (**A**–**H**) Immunofluorescence staining illustrating the expression of IL-1β, IL-18, IL-6, and TNF-α in the synovium of the knee joint of mice, along with quantification of expression. DAPI stains the nuclei blue, and white arrowheads indicate positively stained cells. Data are presented as mean ± SD. ns indicates no statistical significance. * *p* < 0.05, ** *p* < 0.01 (vs. 0 mg/kg group); ## *p* < 0.01 (vs. C57BJ/6 group), with *n* = 6 per group.

As previously demonstrated through HE and MASSON staining, the synovial inflammatory response in SLE mice exposed to MPS is exacerbated. Therefore, we measured the expression levels of inflammatory cytokines using IF. The results showed that exposure to 5 mg/kg MP significantly increased the production of all these inflammatory substances, including IL-1 β, IL-6, IL-18, and TNF-α. However, exposure to 0.5 mg/kg MP did not show statistical changes in IL-6 and TNF-α expression. Obviously, exposure to MP exacerbates synovial inflammation in a dose-dependent manner (Figure 3A–H).

### 3.4. MPs Aggravate Synovial Pyroptosis in Mice with SLE

**Figure 4:**
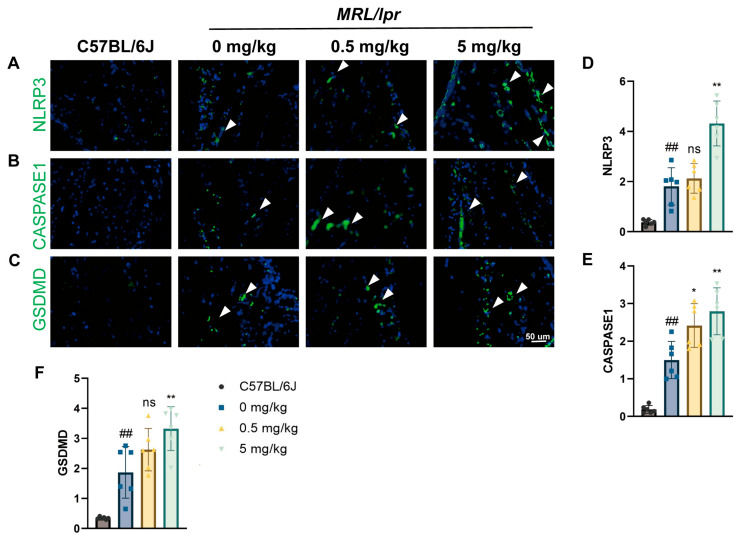
MPs aggravate synovial pyroptosis in SLE mice. (**A**–**C**) Immunofluorescence staining showing the expression of NLRP3, CASPASE-1, and GSDMD in the synovium of the knee joint of mice, along with (**D**–**F**) quantification of expression. DAPI stains nuclei blue, and white arrowheads indicate positively stained cells. Data are expressed as mean ± SD. ns indicates no statistical significance. * *p* < 0.05, ** *p* < 0.01 (vs. 0 mg/kg group); ## *p* < 0.01 (vs. C57BL/6J group), with *n* = 6 per group.

Due to the substantial induction of inflammatory elements in the synovial tissue resulting from MP treatment, we investigated synovial apoptosis by assessing the expression of key proteins involved in NLRP3 inflammasome-mediated pyroptosis, specifically NLRP3, CASPASE-1, and GSDMD. In alignment with previously reported expression levels of IL-1β and IL-18, we found that MP treatment increased the expression of these proteins in a dose-dependent manner within the synovium. However, no significant differences in GSDMD and NLRP3 expression were observed in the 0.5 mg/kg group, as illustrated in Figure 4A–F. Based on these findings, we hypothesize that NLRP3 inflammasome-mediated pyroptosis contributes to synovial damage in SLE mice exposed to MPs.

### 3.5. Effects of MPs Exposure on Oxidative Stress and the NF-κB Signaling Pathway in SLE Mice

**Figure 5:**
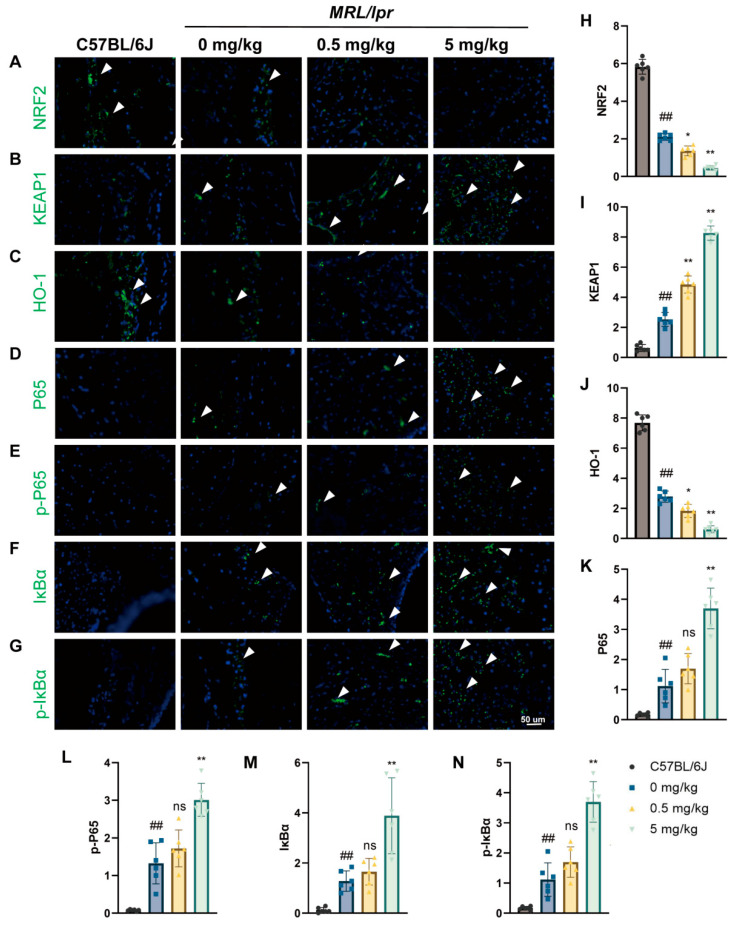
Effects of MPs exposure on oxidative stress and the NF-κB signaling pathway in SLE mice. (**A**–**C**) Immunofluorescence staining was conducted to assess the expression of NRF2, KEAP1, and HO-1 in the synovium of the knee joints of mice, while (**H**–**J**) illustrates the quantification of these expressions. (**D**–**G**) Immunofluorescence staining for P65, P-P65, IκBα, and p-IκBα in the knee joint synovium of mice is presented, with (**K**–**N**) showing the corresponding quantification. DAPI was used to stain nuclei in blue, and white arrowheads indicate positively stained cells. Data are expressed as mean ± SD. ns indicates no statistical significance. * *p* < 0.05, ** *p* < 0.01 (vs. 0 mg/kg group); ## *p* < 0.01 (vs. C57BL/6J group), with *n* = 6 per group.

To elucidate the molecular mechanisms by which MPs aggravate synovial cell infection and pyroptosis in SLE mice, we analyzed the expression levels of NRF2, KEAP1, P65, p-P65, HO-1, IκBα, and p-IκBα to assess the NF-κB signaling pathway and oxidative stress in the synovium. Immunofluorescence was employed to detect oxidative stress and the NF-κB signaling pathway. As shown in Figure 5A–C,H–J, exposure to MPs increased the expression levels of KEAP1 while decreasing the expression levels of NRF2 and HO-1 compared to SLE mice. Additionally, as illustrated in Figure 5D–G,K–N, the expression and phosphorylation levels of proteins related to the NF-κB signaling pathway also increased in a dose-dependent manner following MPs exposure. In summary, these findings suggest that MPs may enhance synovial cell inflammatory responses and pyroptosis by modulating the NF-κB pathway, thereby exacerbating structural and functional damage to the synovium.

## 4. Discussion

MPs are widely distributed pollutants in the environment and organic communities. Sources of MPs include contaminated food, beverages, and plastic products, such as packaging and cooking utensils [23]. MPs can infiltrate the seeds, roots, stems, and leaves of fruits and vegetables, facilitating their entry into the food chain. They enter the human body through various pathways, accumulating within different tissues [23]. Previous research on MPs has primarily focused on vital organs, including the heart, liver, lungs, and reproductive systems. However, there has been limited investigation into the musculoskeletal system, particularly the synovium. In a study by Chang et al. using a rheumatoid arthritis (RA) mouse model, it was found that MPs aggravated knee joint RA by affecting synovial cell proliferation, migration, and inflammation [24]. Our study corroborates these findings, as we observed increased inflammation and cell apoptosis in the knee joint synovium of SLE mice after exposure to 5 μm MPs over nine weeks. The specific pathway through which MPs enter the mouse knee joint and impact synovial tissue morphology and function remains challenging to elucidate. Nevertheless, previous research has suggested that MPs can be transported via capillaries to the ovaries [14,25]. In another study, fluorescent MPs were used to demonstrate that after intravenous injection in mice, MPs were present not only in various organs but also in the knee joints and claws [24]. Therefore, we hypothesized that MPs, following ingestion, are transported to the mouse synovial tissue through capillaries, causing damage to the synovial tissue and exacerbating arthritis in SLE mice.

Evidence indicates that induced oxidative stress in the synovium may play a detrimental role in arthritis by influencing synovial cell inflammation and joint cartilage destruction [26,27]. Moreover, the literature suggests that induced oxidative stress serves as a primary toxic mechanism of MPs in both in vivo and in vitro studies. One study proposed that dietary and water exposure to MPs induces oxidative stress in carp [28], while Wu et al. revealed that MPs can trigger oxidative stress in human Caco-2 cells [29]. To gain a deeper understanding of the mechanisms through which MPs affect the synovium in systemic lupus erythematosus (SLE) mice, we investigated changes in oxidative stress-related factors. Our results indicate that MP exposure decreases the levels of NRF2 and HO-1 in the synovial cells of SLE mice, while increasing the expression of KEAP1. This finding suggests that MPs induce oxidative stress, impair antioxidant capacity, and aggravate oxidative damage in the synovial tissues of SLE mice.

NF-κB is widely involved in cellular transcription processes and participates in various biological activities [30,31]. It has been found that when cells experience oxidative stress, NF-κB can be phosphorylated into phosphorylated-NF-κB [32]. Subsequently, NF-κB activates the transcription of precursor genes, such as IL-1β and IL-18, with these precursor proteins being cleaved into mature forms by CASPASE-1 during pyroptosis [33]. One of the functions of p-NF-κB is to activate the NLRP3 inflammasome [34,35]. As a member of the Nod-like receptor (NLR) family, NLRP3 activates the inflammasome and triggers cell pyroptosis [36]. In our study, we observed accelerated inflammatory responses and increased levels of apoptosis in the synovial tissue of SLE mice following MPs exposure. Simultaneously, the expression of pyroptosis-related elements increased alongside elevated NF-κB protein expression and phosphorylation levels. These findings suggest that exposure to MPs aggravate synovial inflammation and pyroptosis in SLE through activation of the NF-κB signaling pathway, potentially triggered by oxidative stress.

However, this study has several limitations. First, it conducted in vivo experiments and lacked in vitro mechanistic research. Second, it is unclear whether discontinuing exposure to MPs can reverse the synovial toxicity caused by MPs. These will be the directions that we will explore in the future.

The impact of microplastics on various tissues and organs throughout the human body, including joints, is significant. We advocate for a reduction in the use of plastic products in our daily lives.

## 5. Conclusions

In conclusion, our findings demonstrate that SLE mice exposed to MPs experience progressively disrupted antioxidant homeostasis with increasing MPs doses. This disruption manifests as both structural and functional impairment of the synovial membrane, leading to synovial damage. Furthermore, MPs can interfere with antioxidant signaling pathways, including NRF2/KEAP1 and NF-κB, which trigger NLRP3-mediated pyroptosis. This cascade leads to a significant release of inflammatory cytokines, exacerbating damage to the synovial tissue in SLE mice. This study offers novel insights into synovial damage associated with MPs exposure.

## Figures and Tables

**Table 1:** Four criteria for synovial scoring in each group.

	Inflammatory Cell Infiltration	Fibrous Tissue Hyperplasia	Synovial Cell Hyperplasia (Layer Number)	Proliferation of Blood Vessels
0	No	No	No hyperplasia (1 to 2 layers)	No
1	Sparse and scattered	Small	Small (3–4 layers)	Small
2	More	Moderately	Moderately (5–7 layers)	Moderately
3	Massive diffuse	Massive	Massive (≥8 layers)	Massive

## Data Availability

Data available on request from the authors. The data that support the findings of this study are available from the corresponding author, Wei Zhuang, upon reasonable request.
